# Clinimetric properties of the Turkish translation of a modified neck disability index

**DOI:** 10.1186/1471-2474-13-25

**Published:** 2012-02-21

**Authors:** Nur Kesiktas, Emel Ozcan, Howard Vernon

**Affiliations:** 1 IMAE Education and Research Hospital, I. Avrupa Konutları 8. Blok 27 no Atakent mahallesi Kucukcekmece Halkalı, Istanbul, Turkey; 2 Istanbul Faculty of Medicine, Millet Cad. Capa, Istanbul, Turkey; 3 Canadian Memorial Chiropractic College, 6100 Leslie Street, Toronto, Ontario, Canada M2H 3J1

**Keywords:** Neck disability index, Reliability, Validity, Neck pain

## Abstract

**Background:**

Neck pain is a common problem that can greatly affect a person's activities of daily living. Functional status questionnaires are important in assessing this effect, and are used to follow up neck pain management programs. The Neck Disability Index (NDI) is the first-created scale for neck pain-related disability and is widely translated and in common used in many countries. Our aim is investigate to clinometric properties of a Turkish version of modified NDI and to give a choice in daily practise of versions to be used.

**Methods:**

The modified NDI was applied to 30 patients for reliability. 185 patients participated in the validity study. All patients were recruited from the outpatient clinic of our department. The scale was translated by the forward and backward translation procedure according to the COSMIN criteria. The test was repeated at 48 hours interval for reliability study. SPSS-10.0, software was used for statistical analyses. The Intraclass correlation coefficient was used for the test- retest reliability of the modified NDI. Cronbach α was used for internal consistency. Factor analysis was used for construct validity. The validity of the modified NDI with respect to the SF-36, HAD, VAS pain, VAS disability was assessed using Spearman correlations.

**Results:**

The Intraclass correlation coefficient between first and second (within 48 hours) evaluation of test (rs) was 0.92. Questions 1,4,6,8,10 were shown to have excellent reliability. (rs > 0.9). Question 10 was the most frequently challenged question because "recreational and social activities" do not have not the same meanings in Turkey than in western countries. This required that detailed explanations be provided by the investigators. Cronbach's alpha for the total index was 0.88. A single factor accounting for 80.2% of the variance was obtained. Validity studies demonstrated good and moderate correlations (rs) among NDI, HAD, VAS, physical function subtitle of SF 36 (0.62, 0.76, 0.68).

**Conclusions:**

The modified NDI-Turkish version is a reliable and valid test and is suitable for daily practise.

## Background

Neck pain is a common condition [1-8]. It becomes chronic at the rate of 30-50%, thus representing one of the most important reasons of disability and workforce loss [3,4,6,8]. Neck pain has been shown to affect a person's activities of daily living [9-14]. Chronic neck pain results in greatly increased treatment costs and as well as decreases in work capacity. As such, it is important in the early diagnosis and follow-up of neck pain to assess a patient's level of self-rated disability.

Various scales have been developed for the evaluation self-rated disability in neck pain patients. The Neck Disability Index (NDI) [15] is the first such scale published. It was modified from the Oswestry Low Back Pain Disability Questionnaire [16] by Vernon and Mior [15]. The NDI is the most widely used scale for evaluating neck pain related disability throughout the world [17], and it has been translated into many languages such as Brazilian Portuguese [18], Greek [19], Chinese [20], Farsi [21], Dutch [22], Korean [23], French [24]. It has also been translated and used in studies in Turkey [25] along with a Turkish version of the Neck Pain Disability Index [26,27]; however, neither of these studies employed factor analysis of the translated and modified scales. This is important in cross-cultural studies because there is controversy about the factorial structure of the NDI [28,29], and cultural differences may play a role in the variation observed in different studies.

The aim of the present study was to investigate the clinimetric properties of a Turkish translation of a modified NDI in order to evaluate self-rated disability in patients with neck pain.

## Methods

### Study order

The study was planned in three stages. The first stage consisted of the translation and cultural adaptation, including modification, of the original NDI. The second stage consisted of determining the reliability and internal consistency; in the third stage, the concurrent validity of the Turkish modified NDI was studied. This study was conducted without any knowledge of the work of Aslan et al. [25] which may have been conducted concurrently.

### Turkish adaptation

Before starting the study, permission for translation of the test into Turkish was obtained from Dr. Howard Vernon on October 01, 2004. The translation of NDI was performed using back-forward method [30] and conformed to the COSMIN recommendations [31].

The instrument was first translated into Turkish by two native Turkish speaker translators who were blinded to each other. The translation was examined by the study team (three professors whose main field of interest was lower back and neck pain). The instrument was then translated into Turkish again by a native English- speaking translator. The equivalence between Turkish translations and the original English version of the text was then reviewed by the study team. Problems in practice were determined in a small sample of 10 persons. Modifications were then made according to the findings of this group (See: Results, for modifications).

#### Study group

The NDI validity and reliability studies were performed between August 2005-July 2007 on patients with neck pain consecutively presenting in the Physical Medicine and Rehabilitation Clinic of the Istanbul University, Istanbul Faculty of Medicine. The inclusion criteria included:, males and females who were between 18-65 years old, suffering from neck pain alone or from neck pain radiating to arm, with the duration of pain of over 3 weeks, able to read and sign Turkish. Standard clinical, radiological and laboratory tests were applied for diagnosis. Exclusion criteria comprised the inflammatory conditions, malignancy, neck pain due to myopathy, trauma on the neck, prior neck surgery, serious psychiatric disorder and congenital anomalies.

### Statistical analysis

SPSS 10.0 package software was used for statistical analysis. Demographic data (age, gender, education, employment status, etc.) of all patients were recorded (Table 1). In all cases, missing data were counted and were dealt with by SPSS missing value analysis.

**Table 1:** Demographic and clinic data of participants

	Reliability study (n = 30)	Validity study(n = 185)
Age	38.47 ± 4.43	42.73 ± 6.31

Women	15	100

Men	15	85

Body mass indeksi	26.71 ± 3.2	27.3 ± 2.9

Pain duration (month)	34.7 ± 20	36 ± 22

NDI	36.4 ± 20.1	37.5 ± 22.4

### Study designs

#### Test-retest reliability study

Thirty (30) patients of the total sample described below, who presented with neck pain in Physical Medicine and Rehabilitation Clinic of the Istanbul Faculty of Medicine Hospital between August 2005 - July 2007 and who met study criteria, were selected according to random numbers obtained using Quick Calc-(GraphPad Software) [32]. The final version of the Turkish translation of modified NDI (Additional file 1: Appendix S1) was applied twice within a 42-hour mean interval for the test-retest study. During this period no treatment was administered to patients. The difference between the two scales was assessed using paired student's *t*-test. Intra-class correlation coefficient (ICC) was evaluated using one-sided random effects model (1, 1). Test-Retest: in ICC evaluation, values < 0.4 are considered poor, 0.4-0.75 moderate, 0.75-0.9 good and > 0.9 excellent [33]. Cronbach's α was used for internal consistency analysis. Cronbach's α is interpreted as good above 0.80, as moderate between 0.80-0.70, and as low below 0.70 [34].

Factor analysis was performed on the results from all subjects with use of principal component analysis to extract factors. The retained factors in each scale had eigenvalues > 1. Independent factors were obtained by use of the Varimax rotation method [35].

#### Validity studies

In 185 patients who met the inclusion criteria, VAS-pain [36] VAS-disability '0-100', Hospital Anxiety Depression Scale [37,38] and the SF-36 [39,40] scale were used for validity analyses. Since all parameters were not normally distributed, Spearman's correlation was used. Spearman's correlation values were interpreted as excellent for > 0.91 points, good for 0.90-0.71, moderate for 0.70-0.51, poor for 0.50-0.31, and as few or no relation for < 0.30 [41].

#### Scales

Neck Disability Index (NDI), has a total of 10 sections. Each section has six possible answers. Each item is scored from 0 (no disability) to 5 (complete disability). The total score ranges from 0 (no disability) to 50 (total disability), or, in percentage terms, between 0 and 100. Disability increases with increasing score. Items of the scale are: 'intensity of pain', 'personal care', 'lifting', 'reading', 'headaches', 'concentration', 'work', 'driving', 'sleeping' and 'recreation' [15,17].

#### Visual analog scale (VAS)

This scale was used to evaluate subjective pain intensity. Studies have shown that VAS is a reliable and valuable method. The most important limitation of this method is its focusing to a greater extent on the pain severity, and remaining inadequate in evaluating the affective aspect of pain [36].

Hospital Anxiety and Depression Scale (HAD) is a self-evaluating scale used to determine a patient's risk with respect to anxiety and depression, and to assess the level and change in its severity. It contains total of 14 questions, and seven of these assess anxiety while the other seven measure depression [37,38].

Short Form-36 (SF-36) is a scale for quality of life with generic measurement qualities that provides a comprehensive assessment. The scale is comprised of 36 items which provide measurement of 8 domains: Physical functioning (10 items), social functioning (2 items), role limitations due to physical problems (4 items), role limitations due to emotional problems (3 items), mental health (5 items), energy/vitality (4 items), pain (2 items), general perception of health (5 items). Subscales assess health according to scores ranging from 0-100.0 indicates the worst health status; 100 defines the best health status [39,40].

### Hypotheses

We predicted that the test-re-test and internal consistency coefficients would exceed 0.80. We predicted that there would be a 1-factor structure to the Turkish NDI. We predicted that the correlations between the Turkish-NDI and the pain VAS, HAD and SF-36 mental and physical scales would be moderately large (above 0.50).

### Ethics

The study was approved by the Ethics Committee of the Istanbul University of Istanbul Medical Faculty (numbered 270/04).

## Results

In the translation-pilot study, in the first item on "pain intensity" a modification was made as to "your neck pain". In the third item, 'lifting', a statement of "having weight equal to that you lift when you don't have neck pain" was added for the purpose of providing clarification regarding lifting heavy loads. In item seven, 'work', a statement of "Please check the option G if you are not employed," was added to make it clear that it was the work-life that was evaluated.

In the pilot study, the most frequently asked question by participants was associated with item 10. Since "recreation" was better understood as "leisure time activities", it was modified in this way. In the pilot study, there were 9 persons who were non-drivers, and problems arose in answering item 8. Furthermore, to a lesser extent, since there were participants who were non-workers and who did not perform leisure time activities, the option of "never done" was added to sections 7, 8 and 10. The Turkish version of the modified NDI was also used in validity study and, 120 participants (64%) scored item 8 as "never done".

In general, patient comments were that the questions were clear. The time for completing the Turkish version of modified NDI was 5.5 minutes. When there were questions that were not understood, explanation was provided by the observer.

### Participants

Table 1 shows the clinical characteristics of patients who participated in the test-retest study and of those who participated in the validity study. No statistically significant difference was found between the two groups by student's *t *test (p > 0.05).

### Missing data

There were no missing data in NDI. There were very few missing data but no more than 1 item was left unscored in HAD. Percentage of missing data for item 9 in HAD was 1.5%. Percentage of missing item was 0.1% for HAD. That was not remarkable.

### Test-retest

ICC ranged between 0.87-1.0 for all domains (Table 2). The Turkish version of modified NDI -ICC score was 0.92. There was no significant difference between test-retest scores in paired *t *test (p > 0.05). Internal consistency was found as Cronbach's α: 0.88. Questions 1,4,6,8,10 were shown to have excellent reliability (r's > 0.9). Question 10 was the most frequently challenged question because "recreational and social activities" do not have not the same meanings in Turkey than in western countries. This required that detailed explanations be provided by the investigators.

**Table 2:** Reliability study (ICC)

	ICC	CI%95
Pain intensity	0.90	(0.87-0.93)

Personal care	0.87	(0.85-0.91)

Lifting	0.88	(0.85-0.92)

Reading	0.91	(0.88-0.94)

Headaches	0.87	(0.85-0.91)

Concentration	0.93	(0.89-0.98)

Work	0.89	(0.86-0.92)

Driving	1	

Sleeping	0.89	(0.86-0.92)

Leisure activity	0.93	(0.89-0.97)

### Factor analyses

One factor was extracted in exploratory factor analyses, which accounted for 80.2% of the total variance.

### Validity

A total of 185 patients participated in this study. Except for 'physical role' sub-scoreof the SF 36, significant correlations were found between the Turkish version of the modified NDI and VAS pain (r = 0.6), VAS disability (r = 0.76), HAD depression (r = 0.62), and HAD anxiety (r = 0.58). For the SF-36, there were weak correlations with emotional role and pain, while the strongest correlation (r = 0.68) was with SF-36 physical domain. (Table 3).

**Table 3:** NDI validity study

	Mean ± SD	Correlations with NDI
VAS Pain	61.5 ± 25	0.60*

VAS Disability	51.2 ± 29	0.76**

HAD depression	7.3 ± 4.2	0.62*

HAD Anxiety	9.7 ± 4.5	0.58*

SF-36		

Physical functioning	62.5 ± 24.3	0.68*

Role Physical	31.2 ± 24.3	0.29

Bodily Pain	39.4 ± 21.5	0.42^ϕ^

General health	48.1 ± 18.9	0.55*

vitality	49.2 ± 23.1	0.57*

Social functioning	60.2 ± 18.2	0.64*

Role Emotional	37.1 ± 19.9	0.36^ϕ^

Mental health	57.2 ± 21.2	0.60*

## Discussion

For the purpose of evaluating subjects with neck pain in the Turkish society, a Turkish translation and adaptation of NDI was performed, and the validity and reliability of its use was demonstrated. Our study group for the test which was performed on patients from polyclinic through randomized selection comprised of patients with neck pain. This was a homogeneous population regarding age and gender.

In our study, the duration for completing Turkish version of modified NDI was similar to that in the original study [15,17]. Unclear questions were explained by the observer.

As with other studies, there were non-drivers, unemployed and those who did not have leisure time activities [42,43]. Ackelman et al. modified this part of the index wherein they added the explanation of "not applicable" [44]. It was considered appropriate to add the option of "never done" to the test also in our study.

As with most other studies, [18,19,21,24,42-45] test-retest for all NDI domains was found to be high (0.87-1) in our study. In the study by Ackelman et al. the result (0.97) that was found in the test-retest reliability performed with a 2-day interval was high [44], which was attributed to the additional explanations provided by the investigators. These additions were also provided in our study. The study by Cleland et al., determined an ICC of 0.50, which was the lowest value reported in the literature [46]. However, this study involved patients with cervical radiculopathy. In the adaptation study conducted in the Netherlands, test-retest ICC was found lower as 0.53 in personal care domain [22].

The most important comparison of our results is with the work of Aslan et al. [25]. They also reported very high test-retest reliability (r = 0.98).

As with our study, in most similar studies, a duration of a day or two was given for test-retest [18,19,21,24,42-45]. In pain studies, a retest interval of one week was reported to be not suitable as treatment administration and would be unethical [18].

In NDI internal consistency studies, values were found to be between 0.74-0.93 by investigators. The internal consistency we determined in Turkish version of modified NDI was consistent with those found in the studies by these investigators [18,19,21,23,24,42,45]. Aslan et al. [25] did not report on the internal consistency of their Turkish translation of the NDI.

In our study, factor analyses revealed one dimension. Similar factorial structures of the questionnaire were observed in its Greek, Brasilian, Canadian [47], Spanish versions. But, in French version, two factor were found. The percentage of explanation of the single factor was higher than in the Greek version, but similar to that found in the Brasilian and Canadian versions (84%) [18,47]. Aslan et al. [25] did not report on the factorial structure of their Turkish translation of the NDI.

In validity studies, pain was generally evaluated using VAS. Similarly, the results of our study was found good [21,23,45]. Mousavi et al. found a correlation with VAS as r = 0.71 [21]. In these studies methodological basis was similar. In addition, results are parallel with those found in many studies that followed different methodologies [23,45]. Aslan et al. reported only moderately good (r = 0.51-0.62) correlations with the pain VAS [25].

The SF-36 was used for validity also in other studies [18,19,21,44,45,48]. In the Portuguese translation study conducted in Brazil, no correlation was found with physical role, emotional role and pain subtitles of SF-36 [18]. Similarly, no correlation was found with physical role in our study, but there was a weak correlation with emotional role and pain. This could be associated with the number of participants. Riddle et al. found equivalence at strong correlations with physical and mental parts of SF-36 [48]. In the translation study for the scale conducted in Iran, only emotional role was found not to be correlated [21]. This result also supports our study.

The HAD test was used in the translation study conducted in France. Investigators, who found correlation between HADS depression scores and NDI, stated that sense of pain was closely associated with psychology [24]. In our study, HADS was found to be correlated with both anxiety and depression scores. In studies conducted on patients with neck pain, it was shown that anxiety and stress might either be the cause or the result of neck pain [49,50].

In the comparison with Aslan et al., [25] no correlations with self-rated questionnaires for any other important health-related variables was reported. They did report a high correlation with the Turkish version of the Neck Pain Disability Index, which would be expected.

With respect to the work of Aslan et al. [25], in summary, our study has extended that work by including analyses of internal consistency and factorial structure as well as analyses of convergent validity with the pain VAS, HAD and SF-36.

This study has limitations. As this was a study of a modified version of the Turkish NDI, an Item Response Theory (IRT) approach [31] was not adopted; however, with regard to the reliability study, individual item analyses were undertaken. With regard to validity, only current pain, anxiety/depression and quality of life were assessed for concurrent validity. This was deemed to be an appropriate profile of separate constructs with which to evaluate the modified version of the Turkish NDI. Most of the correlations with these instruments did conform to our moderately high predictions. Additional testing with other constructs such as catastrophization or fear-avoidance beliefs would be interesting.

An additional limitation was the lack of assessment of measurement error and responsiveness. We intend to pursue this is a separate study involving a treatment phase.

## Conclusion

The modified NDI-Turkish version has been found to be a reliable and valid test and is suitable for daily practise for characterizing concurrent disability. Its use in determining clinical response has not yet been determined.

## Competing interests

The authors declare that they have no competing interests.

## Authors' contributions

All authors read and appoved the final manuscript. NK participated in study design, pretesting, carried out data entry and interpretation of data and wrote the final draft of the manuscript.EO participated design of the study and translation process. HV provided appraisal and made suggestions during all stages of translation process and participated desingn of study and revised the final manuscript.

## Pre-publication history

The pre-publication history for this paper can be accessed here:

http://www.biomedcentral.com/1471-2474/13/25/prepub

## Supplementary Material

Additional file 1 **Appendix S1**. Turkish version of modified Neck Disability Index. Click here for file
