# Supplementary material for: Clinimetric properties of the Turkish translation of a modified neck disability index
Source: BMC Musculoskelet Disord. 2012 Feb 21;13:25. doi: 10.1186/1471-2474-13-25 (PMC3305639; doi:10.1186/1471-2474-13-25)
Supplement: Additional file 1 — Appendix S1. Turkish version of modified Neck Disability Index. [file 1471-2474-13-25-S1.DOC]

Appendix 1. Turkish version of modified Neck Disability Index

BOYUN ÖZÜRLÜLÜK SORGULAMA FORMU

Bu sorgulama formu boyun ağrınızın günlük yaşam aktivitelerinizi yerine getirme yeteneklerinizi nasıl etkilediğini anlamamıza yardımcı olacak şekilde tasarlanmıştır. Lütfen her bölümdeki bir kutucuğu işaretleyiniz. Bir bölümde birden çok yanıtı kendinize yakın hissetseniz bile, şu anki durumunuza en yakın olan seçeneği işaretleyiniz.

Bölüm 1 - Boyunda Ağrı Yoğunluğu

A- Şu anda hiç boyun ağrım yok.

B- Şu anda çok hafif derecede boyun ağrım var.

C- Boyun ağrım orta derecede ve gelip gidiyor.

D- Boyun ağrım orta şiddette ve değişkenlik göstermiyor.

E- Boyun ağrım şiddetli fakat gelip gidiyor.

F- Boyun ağrım şiddetli ve değişkenlik göstermiyor.

Bölüm 2 - Kişisel Bakım (giyinme ve temizlenme)

A- Ek bir ağrıya neden olmadan kendime bakabiliyorum.

B- Kendime normal olarak bakabiliyorum fakat bu ek bir ağrıya neden oluyor.

C- Kendi bakımımı yaparken ağrım artıyor, yavaşlıyorum ve dikkatli oluyorum.

D- Biraz yardıma ihtiyacım var fakat kişisel bakımımın çoğunu yapabiliyorum.

E- Kişisel bakımım ile ilgili işlerin çoğunda her gün yardıma ihtiyacım var.

F- Giyinemiyorum. Zorlukla yıkanıyorum ve yataktan çıkmıyorum.

Bölüm 3 – Yük Kaldırma (boyun ağrınız olmadığı zamanlarda kaldırdığınız ağır yüklere eşit ağırlıkta)

A- Ek bir ağrı hissetmeden ağır yükleri kaldırabiliyorum.

B- Ağır yükleri kaldırabiliyorum, fakat ek bir ağrıya neden oluyor.

C- Ağrı yükleri yerden kaldırmama engel oluyor, fakat yükler, örneğin masa üstü gibi uygun bir yere yerleştirilirse kaldırabiliyorum.

D- Ağrı ağır yük kaldırmama engel oluyor, fakat hafif ve orta ağırlıktaki yükler örneğin masa üstü gibi uygun bir yere yerleştirilirse kaldırabiliyorum.

E- Çok hafif yükleri kaldırabiliyorum.

F- Hiçbirşeyi kaldıramıyorum ve taşıyamıyorum.

Bölüm 4 - Okuma

A- Hiç boyun ağrısı hissetmeden istediğim kadar okuyabiliyorum.

B- Hafif bir boyun ağrısı hissederek istediğim kadar okuyabiliyorum.

C- Orta derecede boyun ağrısı hissederek istediğim kadar okuyabiliyorum.

D- Boynumda orta derecede ağrı nedeniyle istediğim kadar okuyamıyorum.

E- Boynumda şiddetli ağrı nedeniyle istediğim kadar okuyamıyorum.

F- Boyun ağrısı nedeniyle hiç okuyamıyorum.

Bölüm 5 - Başağrıları

A- Hiç başağrım yok.

B- Sık olmayan hafif başağrılarım var.

C- Orta derecede başağrılarım var.

D- Sık gelen orta derecede başağrılarım var.

E- Sık gelen ağır derecede başağrılarım var.

F- Hemen hemen herzaman başağrılarım var.

Bölüm 6 – Konsantrasyon

A- İstediğim zaman dikkatimi hiç zorlanmadan istediğim kadar toplayabiliyorum.

B- Hafifçe zorlanarak dikkatimi toplayabiliyorum.

C- İstediğim zaman biraz zorlanarak dikkatimi toplayabiliyorum.

D- İstediğim zaman epeyce zorlanarak dikkatimi toplayabiliyorum.

E- İstediğim zaman dikkatimi toplamakta çok fazla zorlanıyorum.

F- Dikkatimi hiç toplayamıyorum..

Bölüm-7 İş (Herhangi bir işte çalışmıyorsanız lütfen G seçeneğini işaretleyiniz)

A- İstediğim kadar iş yapabilirim.

B- Her günkü işlerimi yapabilirim, ama daha fazlasını yapamam.

C- Her günkü işlerimin çoğunu yapabilirim, daha fazlasını yapamam.

D- Her günkü işlerimi yapamam.

E- Herhangi bir işi zorlukla yapabilirim.

F- Hiçbir iş yapamam

G- Hiç yapmadım

Bölüm 8 - Araba Kullanma

A- Boyun ağrısı hissetmeden araba kullanabiliyorum.

B-Boynumda hafif bir ağrı hissi ile istediğim kadar araba kullanabiliyorum.

C- Boynumda orta derecede ağrı nedeni ile istediğim kadar araba kullanamıyorum.

D- Orta derecede bir boyun ağrısı nedeniyle istediğim kadar araba kullanamıyorum.

E- Boynumda şiddetli ağrı nedeniyle güçlükle araba kullanabiliyorum.

F- Boyun ağrısı nedeniyle hiç araba kullanamıyorum.

G- Hiç yapmadım

Bölüm 9 - Uyku

A- Uyku problemim yok.

B- Uykum çok hafif bozuk (bir saatten az süreyle biraz bozuk).

C- Uykum hafif bozuk ( 1-2 saat uykusuzluk).

D- Uykum orta derecede bozuk (2-3 saat kadar süren uykusuzluk).

E- Uykum çok bozuk (3-5 saat süreyle uykusuzluk).

F-Uykum tamamen bozuk (5-7 saat süresince uykusuzluktur).

Bölüm 10 – Boş zaman aktiviteleri

A- Tüm boş zaman aktivitelerine boynumda ağrı hissetmeden katılabiliyorum.

B- Tüm boş zaman aktivitelerine boynumda biraz ağrı hissederek katılabiliyorum.

C-Boynumdaki ağrı nedeni ile tüm boş zaman aktivitelerinin bir kısmına katılabiliyorum.

D-Boynumdaki ağrı nedeni ile boş zaman aktivitelerinin çok az bir kısmına katılabiliyorum.

E-Boynumdaki ağrı nedeni ile boş zaman aktivitelerine hemen hemen hiç katılamıyorum.

F- Hiç bir aktiviteye hiç bir şekilde katılamıyorum.

G- Hiç yapmadım

“Modıfıed with permission of author, 2004”

Hasta Adı ____________Tarih ____________
